# Antibiotic use during pregnancy: a retrospective study of prescription patterns and birth outcomes at an antenatal clinic in rural Ghana

**DOI:** 10.1186/s40545-017-0111-0

**Published:** 2017-08-09

**Authors:** Kwesi Boadu Mensah, Kwame Opoku-Agyeman, Charles Ansah

**Affiliations:** Department of Pharmacology, Faculty of Pharmacy and Pharmaceutical Science, Kwame Nkrumah University of Science and Technology, Kumasi, Ghana

**Keywords:** Pregnancy, Birth outcomes, Congenital defects, APGAR scores, Antibiotic, Intrapartum, Birthweight

## Abstract

**Background:**

Babies are increasingly being exposed to antibiotics intrapartum in the bid to reduce neonatal and maternal deaths. Intrapartum antibiotic exposure, including even those considered safe in pregnancy, have been associated with childhood obesity and compromised immunity. Data on the extent of antibiotic use, safety and its impact on birth outcomes and neonatal health in Sub-Saharan Africa is very limited. This study sought to ascertain the extent of antibiotic use in pregnancy and its effects on birth outcomes in a rural hospital in Ghana.

**Methods:**

The study was a retrospective randomized study of mothers who delivered babies in a rural hospital between 2011 and 2015 in Ghana. A total of 412 mother/baby records out of 2100 pre-selected met the inclusion criteria of the study. Indicators of neonatal health used were birthweight, Apgar score, incidence of birth defects.

**Results:**

Sixty five percent of pregnant women were administered antibiotics at some stage during pregnancy. Beta Lactam antibiotics accounted for more than 67% of all antibiotics prescribed. There was a statistically significant association between antibiotic exposure and pregnancy factors such as stage of pregnancy, parity and mode of delivery but not with socio-economic status of the mother. Intrapartum antibiotic exposure did not significantly affect the birthweight, incidence of congenital birth defect and mean Apgar scores. After adjusting for method of delivery, however, perinatal antibiotic use (24 h to delivery) was associated with lower mean Apgar scores. Birth weight was affected significantly by maternal socio-economic factors such as age and marital status.

**Conclusion:**

Sixty five percent of women attending the antenatal clinic received antibiotics. Intrapartum antibiotics did not affect early markers of neonatal health such as birthweight, congenital birth defect and mean Apgar scores. However, antibiotic use less than 24 h to delivery was associated with a decrease in mean APGAR score.

## Background

There has been a sturdy increase in global antibiotic prescription and consumption [1]. Several concerns have been raised about irrational use of antibiotics and its subsequent impact on drug resistance and cost of healthcare. Furthermore, the role that human microbiota, believed to account for the greater part of human body weight, plays in programming the immunity and metabolism of an individual has re-emphasized the need to regulate use of antibiotics [2].

Pregnancy presents with significant risk of complications which may lead to morbidity and mortality for mother-baby pairs. Globally, more than 50% of neonatal deaths occur in Sub-Saharan Africa because of infections and limited health resources [3, 4]. To manage the complications associated with pregnancy and motherhood, many medicines are employed. Antibiotics remain important in pregnancy and may be second to only iron and food supplement [5]. Due to limited health resources, such as laboratories to carry-out culture and sensitivity test, the extent of exposure of pregnant women and babies to antibiotics and the effects on their health may be underestimated.

There have been several attempts to promote rational antibiotic use partly because of the risk of drug resistance, increased cost of healthcare, as well as the risk of teratogenicity in the developing foetus [6, 7]. However, recent studies have shown that there may be other insidious risk associated with antibiotic use in pregnancy independent on safety profile, mechanism of action or even class the of antibiotic [2]. For instance it is well documented that Caesarian birth or intrapartum antibiotic exposure contributes adversely to childhood obesity and compromised immunity of the baby [8, 9]. Unlike classical teratogenesis, these effects occur indirectly through alterations of the maternal microbiota without the agent necessarily crossing the placenta to affect the tissues of the foetus.

Neonatal Health and Birth outcome is highly dependent on maternal health, nutrition and socio-economic factors. The use and choice of antibiotic during pregnancy depends primarily on maternal factors such as health, nutrition, mode of delivery and socio-economic factors. These maternal factors influence the indicators used to estimate birth outcomes and neonatal health such as birth weight, APGAR scores, and incidences of birth defects. To able to understand the full extent and effects of intrapartum antibiotics on mother and babies, there is a need to elucidate the complex interplay between maternal health, mode of delivery and socio-economic status and neonatal health.

A key point in the millennium development goals is reducing maternal and neonatal mortality. To date there is little clinical data on the trends of antibiotic use during pregnancy and its effects on neonatal health from sub-Saharan Africa. Using Birth weight, APGAR scores, and incidences of birth defects as indicators of birth outcomes and early neonatal health and survival, this study examines the use of antibiotics during pregnancy, its effects on birth and neonatal health and as well as the effects of other maternal factors on antibiotic use in a rural health facility in Ghana.

## Methods

### Study area

Seventh-Day Adventist Hospital, Dominase is a 45 bed capacity hospital located in Dominase about 19 km off the Kumasi–Cape coast highway. The hospital has an obstetrics and gynaecology unit which runs an antenatal clinic with a resident gynaecologist, pharmacist and several midwives and nurses. To limit the impact of un-prescribed and undocumented antibiotic usage, the area chosen was predominately rural with no existent pharmacy. Furthermore, the National Health Insurance Scheme of Ghana runs a “capitation policy” where patients choose a particular primary health care facility for their needs without paying out of pocket. Patients who visit any other facility aside the allocated facility have to pay to access healthcare. This discouraged the tendency for mothers from seeking health services from other facilities as well as reduce self –medication. This is of particular importance because antibiotics can be obtained without prescription in Ghana.

### Study design

The design was a retrospective descriptive study. The primary source of data was prescriptions generated and recorded in patient medical records filed in the hospital between January 2011 and December 2015.

### Sampling method

Folder numbers of all live babies born in the hospital between January, 2011 and September, 2015 were retrieved from the registration book at the maternity unit. Five hundred folder numbers were selected for each of the 5 years from the registration book using systematic sample, making a total of 2500 folders. The contents of 2100 folders were available for the study. Each folder of mother-baby pairs was subsequently screened using the inclusion and exclusion criteria. A total of 412 mother-baby pairs met the selection criteria.

The Inclusion criteria included;

- The mother should have attended at least three ANC at the hospital after confirmation of pregnancy either by ultrasonography or Human Chorionic Gonadotrophic (HCG) detection method.
- Should have delivered a live singleton baby at the facility.

The exclusion criteria included;

- Twin gestations
- Deliveries of referral cases from other hospitals

### Statistical analysis

Data obtained from the medical records were transferred unto a data capture form, the secondary data was later collated, scrutinized and analysed using IBM Statistical package for Social Sciences (SPSS) version 21. Pearson Chi-square test was used to analyze categorical variables. Relative risk and Odds ratio were used as a measure of the degree of association between an event (outcome) and comparable groups. Students T- test and One way analysis of variance (one-way Anova) were used to analyze the difference in mean values between a set of comparable groups or values. Bonferroni was used as the post hoc test after one- way Anova. A *p*-value less than 0.05 was considered statistically significant.

### Potential confounders

The study assumed that antibiotic prescribed was synonymous with exposure which is not always the case. Because of the rural nature of the setting the hospital dispensary dispenses most of the medications prescribed at the same facility. This reduces the inconvenience of patients moving to city centers for their prescribed medicines. The dose prescribed, the duration of therapy and the frequency of administration were not taken into consideration.

The design of the study could not totally account for possible out-of- hospital antibiotic use. The choice of a rural community with few chemical shop outlets and no pharmacy was expected to reduce this potential confounder.

The National Health Insurance Scheme runs a “capitation policy” where a patient chooses one primary health care facility for their needs. The policy also reduces patients moving from one hospital to another because if a patient visits another hospital they have to pay hospital charges. This reduces attrition and tendency for self- medication.

## Results

### Sociodemographic data

The biodata recorded were mothers’ age at birth, gravida, marital status, occupational status and religious affiliation. Mothers’ age at birth ranged from 13 to 44 years with a mean age of 26.3 ± 6.40 years. Most of the pregnant women were between 20 and 30 years, married and average number of gravida of 3.07 ± 2.03. (Table 1).

**Table 1:** Socio-demographic characteristics of participants

		Frequency	Percent
Marital status	Married	304	82.2
	Single	66	17.8
Mothers age at birth	≥19	56	13.7
	20–30	251	61.4
	≤31	102	24.9
Occupation	Farmer	126	34.3
	Trader	78	21.3
	Student	43	11.7
	Teacher	8	2.2
	Seamstress	27	7.4
	Hairdresser	51	13.9
	Nurse	3	.8
	Unemployed	27	7.4
	Others^a^	4	1.0
Religious affiliation	Christian	333	91.2
	Muslim	24	6.6
	Traditional	8	2.2
GRAVIDA	1–3	212	68.8
	≤ 4	96	31.2
	Total	412	100.0

^a^Others include sprayer, baker, secretary and army officer

### Prevalence of antibiotic exposure

About two out of three women (65.8%, *n* = 271) attending ANC clinic in the hospital were treated with an antibiotic at a point during pregnancy. There was no association between the socio-economic status of a woman, i.e., marital status, occupation, age etc. with the odds of being treated with antibiotics. However antibiotic use was associated multiple gravida and Cesarean section. The odds ratio for antibiotic exposure during Caesarean were 13.8 times higher (95% CI, 5.9–32.5, K = 55.47) than that for women who delivered vaginally.

**Fig. 1:**
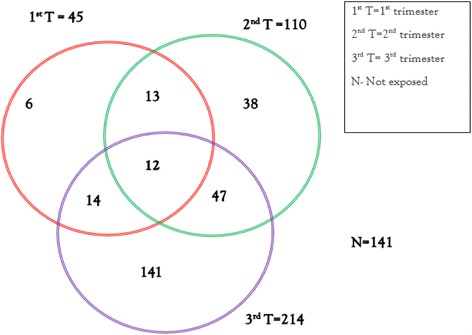
Antibiotic use during pregnancy during the different stages of pregnancy**.** 12 respondents received antibiotics at all stages of pregnancy. 141 did not received antibiotics at all. 13 received during the 1st and 2nd trimesters only. 47 received antibiotics during the 2nd and 3rd trimesters only. 14 respondents received during the 1st and 3rd trimesters only. 141 respondents were treated during the 3rd trimester

There was an association (*p* < 0.001) between antibiotic exposure with advance in stage of pregnancy (Fig. 1). Most exposures (79.0%) were third trimester exposures. In fact 42.4% all antibiotic treatments occurred within 24 h to delivery and about 84% of these women went through Caesarean section. First trimester exposures accounted for just 16.6%. Furthermore 4.40% women received antibiotics in all three trimesters. Interestingly, 5.5% went through gestation and Caesarian without receiving any antibiotics at all (Table 2).

**Table 2:** Antibiotic exposure in pregnant women of different socio economic class and mode of delivery

Antibiotic exposure					
		Yes	No Total		Statistics
Marital status	Married	197(64.8%)	107(35.2%)	304	*P* = 0.77,DF = 1,
	Single	44(66.7%)	22(33.3%)	66	k = 0.83,OR = 0.97
Age of mother at birth	≥ 19	32(57.1%)	24(43.9%)	43	*P* = 0.357, DF = 2
	20–30	167(66.5%)	8(33.5%)	238	K = 2.058
	≤ 31	69(67.6%)	33(32.4%)	96	
Occupation	Employed	182(64.6%)	105(37.4%)	126	*P* = 0.61, K = 10.06
	Student	27(62.8%)	16(37.2%)	43	
	Unemployed	19(70.4%)	8(29.6%)	27	
Religion	Christian	219(65.8%)	114(34.2%)	333	*P* = 0.368, K = 1.99
	Muslim	15(62.5%)	9(37.5%)	24	DF = 2
	Traditional	5(62.5%)	3(37.5%)	8	
GRAVIDA	1–3	117(60.9%	75(39.1%)	192	***P*** **= 0.066**, K = 3.38
	≤4	67(72.0%)	26(28.0%)	93	DF = 1 OR = 0.83
Method of delivery	C. SectioN	104(94.5%)	6(5.5%)	110	***P*** **< 0.001**,K = 55.47
	Vaginal	167(55.3%)	135(44.7%)	302	DF = 1 OR =13.8
Total		**271(65.8%)**	**141(34.2%)**	**412**	

Prevalence of antibiotic use in pregnancy showed a steady increase of 54.8% in 2013 to a peak of 77.8% in 2015.

### Classes of antibiotics prescribed

**Fig. 2:**
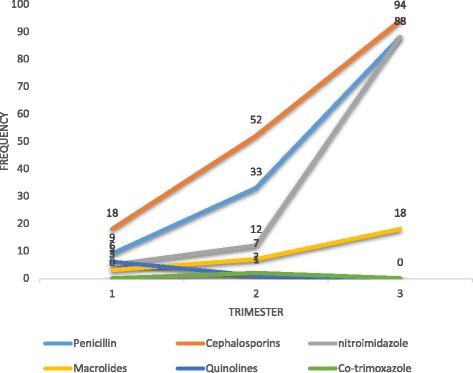
Classes of antibiotic use and the trimester of exposure

Beta lactams antibiotics i.e. cephalosporins and penicillins accounted for more than 67% of antibiotics used whilst metronidazole was used in 24% of pregnant women. Quinolones and sulphonamide/trimethoprim represented about of 2.1% antibiotics prescribed. Cephalosporins, penicillins and metronidazole showed a steep rise in use from first trimester to third trimester (Fig. 2). Macrolide use diminished as pregnancy progressed. Use of quinolones and co-trimoxazole however declined to zero in third trimester. 60% of the patients were given antibiotic because of a urinary tract infection, 12% due to respiratory tract infections, 14.3% as premedication for caesarean section. Other minor reasons included gastroenteritis (5.5%), premature rapture of membranes (2.8%), pelvic inflammatory disease (1.7%) and unspecified indications (3.7%).

### Safety and appropriateness in pregnancy

The FDA classifications for the drugs prescribed in the study fell into classes B, C and D with classes A and X antibiotic not prescribed. Most of the drugs fell into category B with 96.6% followed by C and D with 2.9% and 0.5% respectively. It was observed that some antibiotics were prescribed without due justification i.e. there was drug –diagnosis mismatch and this constituted 3.5% of all prescriptions issued.

### Intrapartum antibiotic exposure and birth outcomes

#### Antibiotic exposure and birthweight

The mean birthweight of babies who delivered at the facility was 2.96 ± 0.56 kg. Intrapartum antibiotic treatment did not affect mean birthweight. Similarly there was no statistically significant relationship between mode of delivery and birthweight. Generally, there was a direct relationship between mothers’ socio-economic status and birthweight. Babies born of single mothers had lower birthweights compared to those born of married mothers (*p* < 0.001, F = 12.74) (Table 3).

**Table 3:** Effect intrapartum antibiotic exposure on weight at birth

	Birth Weight		
		N	Mean ± SD
Marital status	Married	298	**3.02 ± 0.56** ^***^
	Single	65	2.75 ± 0.53
Age group	≥19	55	**2.75 ± 0.48** ^***^
	20–30	242	2.95 ± 056
	≤31	100	3.10 ± 0.57
Occupational status	Employed	287	**3.00 ± 0.57** ^*^
	Student	43	2.75 ± 0.58
	Unemployed	27	3.00 ± 0.47
Religion	Christian	324	2.98 ± 0.57
	Muslim	23	2.82 ± 0.48
	Traditional	8	2.88 ± 0.52
GRAVIDA	1–3	206	**2.87 ± 0.51** ^***^
	4 And Above	95	3.08 ± 0.57
Mode of delivery	Ceasarian section	103	2.96 ± 0.59
	Vaginal delivery	297	2.96 ± 0.55
Antibiotic exposure	Yes	261	2.96 ± 0.58
	No	139	2.97 ± 0.53

Results represented as mean ± SD. * means *p* < 0.05, ***p < 0.001

#### Antibiotic exposure and Apgar scores

There was no statistically significant relationship between antibiotic prescription during pregnancy and mean Apgar scores (i.e. 8.242 ± 1.3 vs 8.32 ± 1.62 (*p* = 0.414, F = 1.59)). However when mothers were treated with antibiotics in less than 24 h to parturition, there was a statistically significant decrease in mean Apgar than those not treated with antibiotics i.e. (7.86 ± 1.72 vs 8.4 ± 1.30 *p* = 0.002, F = 13.65) after adjusting for the effects of the mode of delivery.

Babies born naturally had mean Apgar scores statistically higher than those born by Caesarean section i.e. 8.42 ± 1.23 vs 7.86 ± 1.87(*p* = 0.003, F = 8.98). Younger mothers, unmarried women and mothers with less gravida had significantly higher mean Apgar scores compared with older (*p* = 0.046), married (*p* = 0.018) and higher gravida (*p* = 0.034) mothers respectively (Table 4).

**Table 4:** Effect intrapartum antibiotic exposure on mean APGAR score

APGAR Scores			
		**N**	Mean ± SD
Marital status	Married	282	**8.26 ± 1.37** ^*^
	Single	62	8.69 ± 0.90
Age groups	≤19	53	**8.60** ± 1.10^*^
	20–30	232	8.41 ± 1.28
	≥31	93	8.10 ± 1.43
Occupational status	Employed	272	8.32 ± 1.32
	Student	41	8.40 ± 1.25
	Unemployed	25	8.48 ± 0.95
Religion	Christian	311	8.33 ± 1.34
	Muslim	21	8.43 ± 1.15
	Traditional	1	9.00 ± 0.00
GRAVIDA	1–3	200	**8.54 ± 1.22** ^*^
	≥4	88	8.20 ± 1.27
Mode of delivery	Ceasarian section	100	**8.03 ± 1.67** ^**^
	Vaginal delivery	281	8.47 ± 1.11
Antibiotic exposure	Yes	248	8.30 ± 1.30
	No	133	8.47 ± 1.26
Perinatal antibiotic exposure	Yes	104	**7.96 ± 1.70** ^**^
	No	277	8.51 ± 1.07

Results represented as mean ± SD. *means *p* < 0.05, **p < 0.01

#### Antibiotic exposure and congenital birth defect

Six birth defects were seen in the study representing 1.5% of all deliveries i.e. Cleft lip, mega cephalous, cleft palate and webbed feet. Intrapartum antibiotic exposure irrespective of trimester of exposure was not associated with any significant risk of birth defect (RR = 1, *P* = 0.97). There was no statistically significant association between marital status (*p* = 0.94), mothers age at birth (*p* = 0.361), occupation (*p* = 0.630), parity (*p* = 0.94) and religion (*p* = 0.49) and birth defects. Females had relatively higher odds ratio of incidence of birth defects than males. i.e. 2.2% vs 1.0% (OR = 2.2, *P* = 0.343, 95% CI).

## Discussion

The study was design to ascertain the prevalence antibiotic prescription among pregnant women attending ANC. About 65.8% of pregnant women were exposed to an antibiotic at some point during pregnancy. This figure was higher than an earlier report of about by 59.9% in the eastern region of Ghana [10] as well as the WHO/International Network for the Rational Use of Drugs figures. Women who went through Caesarean section were more likely to receive antibiotics to control wound infection and endometritis [11, 12].

Risk of antibiotic exposure was highest in the last trimester but it corresponded with the increased incidence of respiratory tract infections, urinary tract infections and gastritis during third trimester. This is of a concern because acquisition of specific foetal immunity begins in the third trimester and it is highly dependent on microbiome which can be altered by antibiotics [9, 13].

Most of the antibiotic used are classified as safe in pregnancy by the FDA. Macrolides though safe, directly stimulate the motilin receptors inducing Gastro-intestinal discomfort which may be undesirable in pregnancy [14, 15]. Anatomically the uterus sits directly on the urinary bladder and so increase in weight of the uterus as pregnancy advances exerts a proportional pressure on the bladder and stomach leading to increased incidence of UTI, gastritis and heartburns [16, 17].

Babies exposed to intrapartum antibiotics had mean weights slightly lower than those unexposed. A study by Vidal et al.*,* (2013) [8] reported a similar pattern and it was further observed that such babies quickly gained much more weight after delivery leading to obesity in childhood. This study also noted a strong association between mother’s socio-economic factors and birthweights as has been reported elsewhere [18–20]. This may be due to economic stability, marital support etc [18, 21, 22]. Babies born to Mothers who received antibiotics less than 24 h to delivery or subsequently had caesarean birth had lower mean Apgar scores much even after adjusting for other confounding factors. This correlates with other reports in which babies born vaginally had much higher Apgar scores at 5 min compared to caesarean born [23]. Older mothers or women with higher gravida had babies with mean Apgar scores lower than younger mothers or women with less gravida.

A total six birth defects (15 per 10,000 live births) were recorded in this study. This figure was similar to other prevalence birth defects reported other authors [24].

## Conclusion

65% of pregnant women received antibiotics at some stage during pregnancy. Antibiotic use did not affect mean birth weight, APGAR score or incidence of birth defects. However, antibiotic use less than 24 h to delivery was associated with a decrease in mean APGAR score.

## Abbreviations

ANC: Ante natal clinic
APGAR: Appearance, pulse, grimace, activity and respiration
FDA: Food and drugs authority
RR: Relative risk
SDA: Seventh-day adventist
